# Dichotomous Responses to Chronic Fetal Hypoxia Lead to a Predetermined Aging Phenotype

**DOI:** 10.1016/j.mcpro.2021.100190

**Published:** 2021-12-24

**Authors:** Stefan Rudloff, Andrea Bileck, Lukas Janker, Nicola Wanner, Nastassia Liaukouskaya, Carsten Lundby, Tobias B. Huber, Christopher Gerner, Uyen Huynh-Do

**Affiliations:** 1 Division of Nephrology and Hypertension, University of Bern and University Hospital Bern, Bern, Switzerland; 2 Department of Analytical Chemistry, Faculty of Chemistry, University of Vienna, Vienna, Austria; 3 University Medical Center Hamburg-Eppendorf, III. Medizinische Klinik und Poliklinik, Hamburg, Germany; 4 Centre for Physical Activity Research (CFAS), Rigshospitalet Section 7641, Copenhagen, Denmark; 5 Faculty of Social and Health Sciences, Section for Health and Exercise Physiology, Inland Norway University of Applied Sciences, Lillehammer, Norway

**Keywords:** chronic hypoxia, intrauterine growth restrictions, fetal programming of adult disease, proteomics, premature aging, CAV1, caveolin-1, CKD, chronic kidney disease, DTT, dithiothreitol, ESRD, end-stage renal disease, FA, formic acid, FDR, false discovery rate, GFR, glomerular filtration rate, IUGR, intrauterine growth restriction, LFQ, label-free quantification, MPO, myeloperoxidase, OXPHOS, oxidative phosphorylation, pPTC, primary proximal tubular cell, SASP, senescence-associated secretory phenotype, SIPS, stress-induced premature senescence

## Abstract

Hypoxia-induced intrauterine growth restriction increases the risk for cardiovascular, renal, and other chronic diseases in adults, representing thus a major public health problem. Still, not much is known about the fetal mechanisms that predispose these individuals to disease. Using a previously validated mouse model of fetal hypoxia and bottom-up proteomics, we characterize the response of the fetal kidney to chronic hypoxic stress. Fetal kidneys exhibit a dichotomous response to chronic hypoxia, comprising on the one hand cellular adaptations that promote survival (glycolysis, autophagy, and reduced DNA and protein synthesis), but on the other processes that induce a senescence-like phenotype (infiltration of inflammatory cells, DNA damage, and reduced proliferation). Importantly, chronic hypoxia also reduces the expression of the antiaging proteins klotho and Sirt6, a mechanism that is evolutionary conserved between mice and humans. Taken together, we uncover that predetermined aging during fetal development is a key event in chronic hypoxia, establishing a solid foundation for Barker’s hypothesis of fetal programming of adult diseases. This phenotype is associated with a characteristic biomarker profile in tissue and serum samples, exploitable for detecting and targeting accelerated aging in chronic hypoxic human diseases.

## Highlights

• Chronic fetal hypoxia induces widespread changes in protein expression.
• Repressed replication and translation constrain the formation of new nephrons.
• Multiple pathways predetermine an aging phenotype already during development.
• Synergy of inflammatory damage, ineffective repair, and altered metabolism.
• Reduced abundance of antiaging proteins klotho and sirtuin 6 in mice and humans.

## Graphical Abstract

**Figure:**
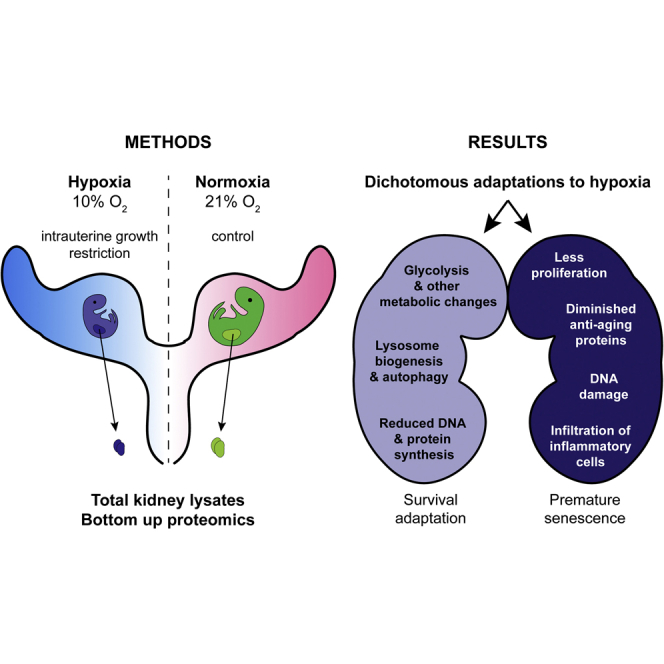

## In Brief

Using bottom-up proteomics and the kidney as a paradigm, we report an integrative perspective of the cellular responses to chronic fetal hypoxia, uncovering fundamental mechanisms of the fetal programming of adult diseases theory that are principally applicable to all other organs in the body. The characteristic tissue and serum biomarker profile will promote the development of novel therapeutic approaches to counteract the premature aging phenotype that seems to be associated with the fetal programming of chronic diseases.

Barker’s theory of fetal programming of adult diseases (1, 2) states that adverse events during development increase the risk of multiple chronic diseases in adulthood, including hypertension, diabetes mellitus, obstructive pulmonary disease, or chronic kidney disease (CKD). In our steadily aging society, these disorders impose an enormous economic burden that is progressively growing due to the increasing number of patients suffering from one or more of these disorders. Among these, CKD and end-stage renal disease (ESRD) not only represent independent risk factors for cardiovascular morbidity and mortality, but also generate increasing costs for renal replacement therapies, making them a major public health problem. Among the strategies to encounter this trend, elucidation of the mechanisms that connect Barker’s theory and the development of CKD play a crucial role. A plausible explanation for the gradual loss of renal function in low birth weight progeny hypothesizes the establishment of a vicious circle revolving between a reduced renal filtration surface, increased work load on the remaining nephrons, and further damage that again decreases the filtration capacity (Brenner’s hypothesis) (3). A common finding in intrauterine growth restriction (IUGR) babies is the reduced number of nephrons formed at the end of kidney development (4). Thus, a low nephron number at birth increases the risk for accelerated functional decline and irreversible renal tissue damage (5, 6). Among all possible intrauterine stress factors, chronic hypoxia due to placental insufficiency or pregnancy at high altitude (>2500 m above sea level—affecting more than 2% of the world’s population (7, 8)) belongs to the most critical and clinically relevant factors to disturb the developmental program of the embryo (9). However, the molecular mechanisms by which hypoxia leads to IUGR and disturbed renal development remain incompletely understood and warrant further investigation. In this study, we assessed on the proteome level the molecular changes in the fetal kidney upon exposure of the developing mouse embryo to chronic hypoxic stress and corroborated some key findings in human hypoxic serum samples. We provide substantial evidence for a range of interacting mechanisms that on the one hand enable the survival of cells in adverse conditions, but on the other hand promote reduced nephron numbers and a premature senescence-like phenotype. We further show that gene-specific DNA hypermethylation but also DNA hypomethylation contribute to this dichotomous response to chronic hypoxia. Thus, our findings establish a solid foundation for Barker’s hypothesis, revealing an array of pathways that predetermine an aging phenotype already during development.

## Experimental Procedures

### Experimental Design and Statistical Rationale

The objective of this explorative study was to uncover changes in the proteome of whole fetal kidneys derived from IUGR fetuses in order to deepen the knowledge about the fetal mechanisms that predispose small for gestational-age individuals to chronic diseases in adulthood. The study was based on bottom-up proteomic profiling using a nano-LC system coupled to a high-resolution orbitrap mass spectrometer. To this end, six E18.5 hypoxic kidneys and six E18.5 normoxic kidneys from males were used and analyzed in duplicate in a controlled experiment in a blinded manner. All replications were successful, no experimental finding could not be replicated. The proteomics data were analyzed using MaxQuant 1.5.2.8 software (including the Andromeda search engine and the Perseus statistical analysis package) and functional annotation clustering. For the long-term consequences of chronic fetal hypoxia on renal function five hypoxic and five normoxic male offspring were chosen randomly and analyzed at 8 months (five animals are required to detect a 30% reduction in GFR in fetal hypoxic animals, based on a variance of 15%, Alpha = 0.05, Power = 0.9). The *in vivo* experiment was concluded with the assessment of renal fibrosis and serum markers after 15 months. To validate some of the key findings of the animal experiments, human serum samples were analyzed, which were collected in previous controlled study of healthy volunteers (10). All *in vitro* experiments were assayed blinded with appropriate controls. The statistical test and experimental details are provided in each figure legend. All data are presented and include all outliers.

### Animals

C57BL/6N mice were purchased from Janvier labs, France, and housed at 23 °C and 50 to 60% humidity in IVC cages with free access to chow and water and a 12 h day/night cycle. All animal experiments were conducted according to the Swiss law for the welfare of animals and were approved by the local authorities (Canton of Bern BE96/11, BE105/14, and BE105/17). For timed mating, females in breeding were checked for vaginal plugs every morning, and if present, the time point was set to gestational day (E) 0.5. Gravid dams were randomly assigned to either a normoxic control group (N = 11) or exposed to constant hypoxia (N = 11) for 7 days. For induction of hypoxia, dams at gestational day E11.5 were transferred into a hypoxic glove box (Coy Laboratory Products), and the oxygen content was gradually lowered to 10% within 3 to 4 h. An electric fan inside the chamber maintained adequate air circulation. The CO_2_ level was kept low by chelating excess CO_2_ in soda lime (Sigma, 72073) filled cartridges connected to the air circulation system. Excess humidity was absorbed by silica gel orange granulate (Sigma, 1.01969), changed every day. Nine dams from each group were euthanized at E18.5, and fetuses and placentas were collected, weighed, and prepared for further analysis. Fetal blood was collected from the trunk of decapitated E18.5 fetuses. Fetal kidneys were dissected in PBS using a Leica M80 stereoscope. For the long-term analyses, two dams were removed from hypoxia a few hours before birth, and the male offspring were used for the evaluation of kidney function (glomerular filtration rate—GFR) at 8 months as described (11) and for the evaluation of renal fibrosis and serum levels of klotho and sirtuin 6 at 15 months.

### Glomerular Count

For whole mount immunofluorescence staining of E18.5 kidneys, the iDisco staining protocol (12) (https://idisco.info/idisco-protocol/) with methanol pretreatment was applied using an antinephrin antibody (R&D AF3159). An incubation time n = 1 day and a solution volume of 1.6 ml were used for the relevant steps. The kidneys were mounted in 8-well glass chamber slides (Thermo Fisher, 154534) and imaged immediately using an IMIC digital microscope (FEI, Type 4001) with a Polychrome V light source, an Orca-R2 camera controller from Hamamatsu (C10600), and Live Acquisition software (FEI, version 2.6.0.14). Hundred-micrometer Z-stack images of stained whole-mount E18.5 kidneys were analyzed with the open-source image processing software Fiji (ImageJ, version 2.0.0-rc69/1.52i, https://imagej.net/Fiji). In the TrackMate v3.8.0 plugin, the Downsample LoG detector was set to 80.0 pixel for the estimated blob diameter with a 16-pixel threshold and downsampling factor 2. The number of spots per frame was added to calculate the number of glomeruli per kidney. A 100 μm distance between frames was chosen to avoid double counting of identical glomeruli in consecutive images, given an average glomerular diameter of 80 μm.

### Transcutaneous Assessment of Glomerular Filtration

The GFR was determined in conscious animals as previously described (11). Briefly, the plasma clearance of FITC-sinistrin (Fresenius-Kabi, LI9830076) is measured across the skin using light-emitting diodes with an emission maximum for FITC at 470 nm and a photodiode detecting the fluorescent light with a maximum sensitivity at 525 nm. The decrease in fluorescence intensity over time is then converted into GFR.

### RT-qPCR

Total RNA was isolated using TRIzol reagent (Invitrogen 15596026) according to the manufacturer's protocol. RNA concentration and quality were determined with a Nanodrop 1000 spectrophotometer (Thermo Fisher Scientific), and 1000 ng was transcribed into cDNA using PrimeScript RT Reagent Kit (Takara, RR037A). cDNA was diluted to 2 ng/μl, and qPCR was performed with FAM-labeled UPL probe (Roche) plus corresponding gene-specific primers and TaqMan Fast Universal PCR Master mix (Applied Biosystems, 4352042) on a 7500 Fast Real-Time PCR System (Applied Biosystems). Data analysis was performed with Microsoft Excel. The 2^(−ΔCt)^ method was used to calculate the relative expression levels for RT-qPCR. Probe and primer sequences are listed in supplemental Table S1.

### Sample Preparation for LC-MS/MS Analysis

Isolated male fetal kidneys (n = 3 for each group) were homogenized in sample buffer (7.5 M urea, 1.5 M thiourea, 4% CHAPS, 0.05% SDS, 100 mM dithiothreitol (DTT)) using an ultrasonic stick. Protein concentrations were determined by means of a Bradford assay (Bio-Rad-Laboratories). An in-gel digestion protocol was applied as described previously (13, 14). In brief, 80 μg of each sample was loaded on SDS-PAGE, which allowed us to prefractionate the sample in order to reduce the complexity thereof. After fixation with 50% methanol/10% acetic acid, the gels were washed and sensitized with 0.02% Na_2_S_2_O_3_. Gels were then stained with 0.1% AgNO_3_ for 10 min, rinsed and developed with 3% Na_2_CO_3_/0.05% formaldehyde. Each protein band was then cut into four slices and destained. Upon reduction with DTT and alkylation with iodoacetamide (IAA), the proteins were digested enzymatically using Trypsin/Lys-C (MS grade; Promega Corporation). Following digestion, the peptides were eluted, dried, and stored at −20 °C until LC-MS/MS analysis.

### LC-MS/MS Analysis

As described previously (13), peptide samples were reconstituted in 5 μl 30% formic acid (FA) containing 10 fmol each of four synthetic standard peptides and diluted with 40 μl mobile phase A (97.9% H2O, 2% ACN, 0.1% FA). Synthetic peptides [Glu1-Fribrinopeptide B – EGVNDNEEGFFSAR; M28 – TTPAVLDSDGSYFLYSK; HK0 – VLETKSLYVR; HK1 – VLETK(ε-AC)SLYVR] were spiked in each sample as internal quality control for monitoring LC-MS-system stability. Ten microliters of this solution was then injected into the Dionex Ultimate 3000 nano LC-system coupled to a QExactive orbitrap mass spectrometer equipped with a nanospray ion source (Thermo Fisher Scientific). As preconcentration step, peptides were loaded on a 2 cm × 75 μm C18 Pepmap100 precolumn (Thermo Fisher Scientific) at a flow rate of 10 μl/min using mobile phase A. Elution of peptides from the precolumn to a 50 cm × 75 μm Pepmap100 analytical column (Thermo Fisher Scientific) and subsequent separation were achieved at a flow rate of 300 nl/min using a gradient of 8% to 40% mobile phase B (80% ACN, 19.9% H2O, 0.1% FA) over 235 min. Mass spectrometric detection was accomplished, performing MS scans in the range from m/z 400 to 1400 at a resolution of 70,000 (at m/z =200). MS/MS scans of the 12 most abundant ions were achieved through HCD fragmentation at 30% normalized collision energy and analyzed in the orbitrap at a resolution of 17,500 (at m/z =200). All samples were analyzed in duplicates. The mass spectrometry proteomics data have been deposited to the ProteomeXchange Consortium (http://proteomecentral.proteomexchange.org) *via* the PRIDE partner repository (15) with the dataset identifier PXD018999 and 10.6019/PXD018999.

### Protein Identification and Label-Free Quantification (LFQ)

Identification of proteins, label-free quantification (LFQ) as well as statistical analyses were performed using the MaxQuant 1.5.2.8 software including the Andromeda search engine and the Perseus statistical analysis package (16, 17), a commonly used workflow for processing and statistical assessment of shotgun proteomics data. Proteins were identified using the UniProt database for mus musculus proteins (version 170428 with 16,854 entries, restricted to reviewed entries only), a peptide mass tolerance of 25 ppm, an MS/MS match tolerance of 20 ppm, and a maximum of two missed cleavages with trypsin as protease. Search criteria further included carbamidomethylation of cysteines as fixed modification, methionine oxidation as well as N-terminal protein acetylation as variable modifications, and a minimum of two peptide identifications per protein, at least one of them unique. Furthermore, match between runs was performed using a 5 min match time window and a 15 min alignment time window. For both, peptides and proteins, a false discovery rate (FDR) of less than 0.01 was applied. For statistical analysis, mutual comparisons between normoxic and hypoxic kidney samples were performed to determine protein groups, which were significantly up- or downregulated upon hypoxia. To this end, using the Perseus statistical analysis package, differences of LFQ values were calculated. Changes in protein abundance values between normoxic and hypoxic kidney samples were determined by a two-sided *t* test with *p* < 0.05 and a minimum of a twofold abundance difference. All proteins meeting these criteria were considered in the present study. In addition, to emphasize the most robust regulatory effects upon hypoxia, significantly regulated proteins with a global FDR <0.05 as determined by a permutation-based method were analyzed.

### Functional Annotation and Clustering

Functional annotation clustering was performed using the gene ontology resource database (18, 19, 20) and the DAVID platform (21) (https://david.ncifcrf.gov). Protein networks were generated using the STRING database (22). Differential expression values between normoxic and hypoxic control group E18.5 kidneys were mapped with Heatmapper (23) (http://www.heatmapper.ca).

### Immunohistochemistry

PFA-fixed, paraffin-embedded tissue sections were rehydrated, and endogenous peroxidase was blocked by incubating the slides in 1.5% H2O2 solution (0.02 M citric acid, 0.06 M Na2HPO4) at RT for 15 min in the dark. Antigen retrieval was performed by boiling in Tris-EDTA (pH 9) or citric acid buffer for 20 min followed by slow cool down to RT. After blocking in 2% BSA in PBS at RT for 1 h, the sections were incubated with primary antibodies in blocking solution o/n at 4 °C (MPO, ab208670; CAV1, ab32577; both Abam). Following three washing steps in PBS, the sections were incubated with HRP-conjugated secondary anti-rabbit antibody (K4003, Dako EnVision+ System from Agilent) for 1 h at RT. After three washing steps in PBS, the signal was developed with DAB (Agilent, K3468). The sections were counterstained, dehydrated, and mounted using Eukitt medium (Sigma, 03989). For the detection of 8-OHdG (MOG-100P, JaICA), the Vector M.O.M immunodetection kit (BMK-2202) was used according to the manufacturer. Bright field images were acquired on a Nikon E600 microscope (Plan Fluor ELWD 20×/0.45 objective) with a Digital Sight DS-UE camera controller and DS-Ri1 camera (both Nikon) using Nikon software NIS Elements 4.0.

### Enzymatic Quantification of Lactate

Lactate levels in E18.5 kidneys were determined after deproteination of the sample with spin columns using the enzymatic kit MAK064-1KT (Sigma), according to the manufacturer.

### Human Studies

The study of human subjects was approved by the Ethics Committee of the Swiss Federal Institute of Technology (EK 2011-N-51) and conducted in accordance with the Declaration of Helsinki. Briefly, informed consent was obtained from, nine healthy, nonsmoking lowlanders (eight males, one female). These volunteers were sequentially assessed for multiple physiological parameters before, during and after a 4-week uninterrupted sojourn at high altitude, as previously reported (10).

### Cell Culture

Primary proximal tubular cells (pPTCs) were isolated from 3- to 4-week-old C57BL/6N male kidneys as previously described (24). Briefly, proximal tubular fragments were obtained by digesting cortical kidney tissue with collagenase and subsequent filtration through a 250 μm and an 80 μm pore size membrane. pPTCs were cultured in DMEM/F12 (Gibco, 21041-025) supplemented with 15 mM HEPES, 0.55 mM NaPyruvate, 1% NEAA, and renal epithelial cell growth medium (REGM) supplements (Lonza, CC-4127). Upon confluency, pPTCs were split once using Accutase solution (Sigma, A6964) and replated. For hypoxia, cell culture was performed at 1% oxygen for 48h; normoxic controls were cultured at 19% oxygen.

### ELISA

Serum levels of Klotho or Sirt6 were determined using the following kits for mouse samples (CSB-E14362m, Cusabio technology and OKCD02892, Aviva Systems Biology) and human samples (CSB-E17018h and CSB-E13235h; both Cusabio technology) following the provided protocols. Mouse fetal blood was collected from the trunk of decapitated E18.5 fetuses. Mouse blood from adult animals was sampled from the vena saphena. Human serum samples were obtained from a previous study (10).

### Bisulfite PCR

DNA from E18.5 hypoxia or normoxia treated animals was extracted by Qiagen Blood and tissue kit and bisulfite-converted using EpiTect Bisulfite Kit from Qiagen according to the manufacturer’s instructions. Twenty nanograms of converted DNA was used as template in a PCR reaction with AmpliTaq Gold (Invitrogen). Following primers were designed using MethPrimer 2.0: Klotho, 248 bp fragment (−234/+14 of TSS), Kl_BS_F (TAG TTT TAG GAA GGT AAA GGG AGT G) and Kl_BS_R (AAC AAT AAT TAT CCA AAA CAA AC) (25); Mki67, 497 bp fragment (∼1 kb upstream of TSS), Mki67_BS_F (TTG GAA GAA TAT TGA TTT AGG AA), and Mki67_BS_R (ACC TAT AAA CTC CCT CTC CAA AAA T). PCR products were ligated into a pCR4-TOPO-Vector using the TOPO TA Cloning Kit for sequencing (Invitrogen) and transformed into DH10 bacteria. Clones were sent for sequencing (Microsyth Seqlab GmbH). Inspection, alignment, visualization, and statistics were performed with QUMA quantification tool for methylation analysis (26).

### Statistics

Statistical analysis were carried out and graphs were made with GraphPad Prism version 8 (https://www.graphpad.com). Two groups were tested for significance by unpaired two-tailed *t*-tests with or without Welch’s correction (Welch’s correction was for nonnormally distributed data, F test of variances was statistically significant, *p* < 0.05). Multiple groups were tested using RM one-way ANOVA with the Geisser–Greenhouse correction and Dunnett’s multiple comparisons test. *p* values <0.05 were assumed significant. Means with standard deviation (SD) are shown.

### Source Data

**Fig. 1:**
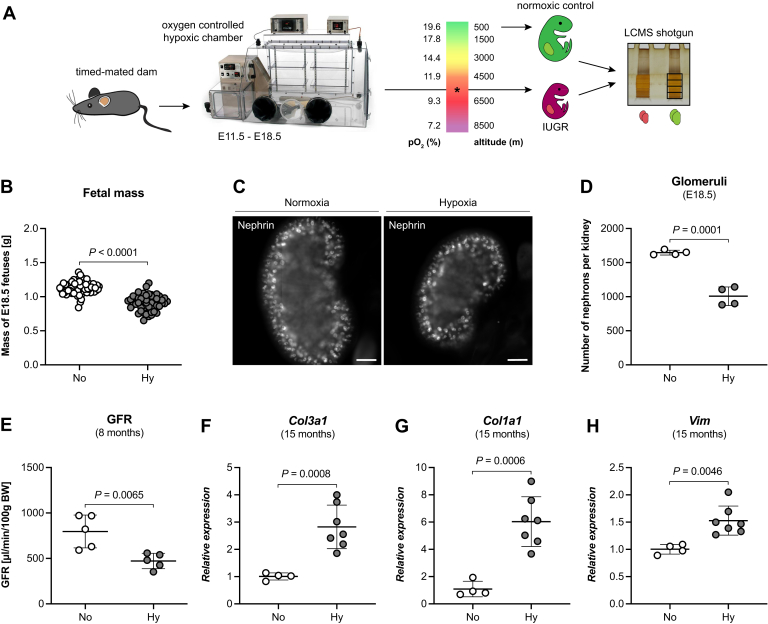
**Chronic fetal hypoxia causes intrauterine growth restriction and impaired renal function in adult offspring.***A*, outline of experimental procedure. Dams at gestational day (E) 11.5 were transferred into an oxygen-controlled hypoxic chamber, and the oxygen was gradually lowered to 10%. After 7 days, fetal kidneys were collected and subjected to mass spectrometry. *B*, weight distribution of E18.5 normoxic (N = 59) or hypoxic fetuses (N = 52) (unpaired two-tailed *t* test, *p* < 0.0001). *C*, the number of nephrons (glomeruli) in hypoxic E18.5 kidneys was significantly reduced (unpaired two-tailed *t* test, *p* = 0.0001). *D*, representative fetal kidney sections stained for the glomerular marker Nephrin showing the reduced number of nephrons quantified in (*C*) (Scale bar = 100 μm). *E*, this lower nephron number at birth resulted in a reduced glomerular filtration rate (GFR) in adult mice at 8 months (unpaired two-tailed *t* test, *p* = 0.0065; for No: 797 ± 161 μl/min/100 g BW, for Hy: 472 ± 76 μl/min/100 g BW; post-hoc power = 0.93, Alpha = 0.01). *F*–*H*, furthermore, at 15 months hypoxic offspring had significantly higher mRNA expression levels of the fibrotic markers vimentin (*F*, Vim, unpaired two-tailed *t* test, *p* = 0.0046), type I collagen (*G*, Col1a1, unpaired two-tailed *t* test, *p* = 0.0006), and type III collagen (*H*, Col3a1, unpaired two-tailed *t* test with Welch’s correction, *p* = 0.0008) than normoxic aged-matched controls.

The raw data for all experiments that do not result from the mass spectrometry analysis can be found in the spread sheet Source Data File S1 in the Supplementary data set. This includes data underlying Fig. 1*A* and *D–H,* Fig. 3*E* and *F,* Fig. 5*B,* Fig. 7*D–I,* Fig. 8, and supplemental Fig. S1 and Fig. S6.

## Results

### Chronic Fetal Hypoxia Results in Growth-Restricted Embryos and Reduced Renal Function in Adult Offspring

To expose timed-mated dams to constant hypoxia (10% oxygen) for 7 days (Fig. 1*A*), a hypoxic chamber was used. Similar to our previous work (27), dams quickly adapted to the hypoxic environment, and at birth litter size and placental mass were similar to normoxic control gestations (supplemental Fig. S1, *A*–*C*). Nevertheless, hypoxic E18.5 fetuses had a reduced body weight compared with normoxic controls, fulfilling IUGR criteria (28) (Fig. 1*B* and supplemental Fig. S1*D*), and a 40% reduced nephron number (Fig. 1, *C* and *D*). This reduction was not due to nephron degeneration (necrosis or apoptosis) during intrauterine development under hypoxic conditions as previously reported (27). Furthermore, despite a considerable catch-up growth period 3 weeks after birth, which we reported before (27), adult hypoxic offspring showed severely reduced renal function at 8 months (Fig. 1*E*) and increased expression of fibrotic markers at 15 months (Fig. 1, *F*–*H*), thus confirming the validity of our approach as a robust IUGR model and illustration of Barker’s theory of fetal programming of adult diseases.

### Hypoxic Fetal Kidneys Exhibit a Deregulated Protein Expression Profile

**Fig. 2:**
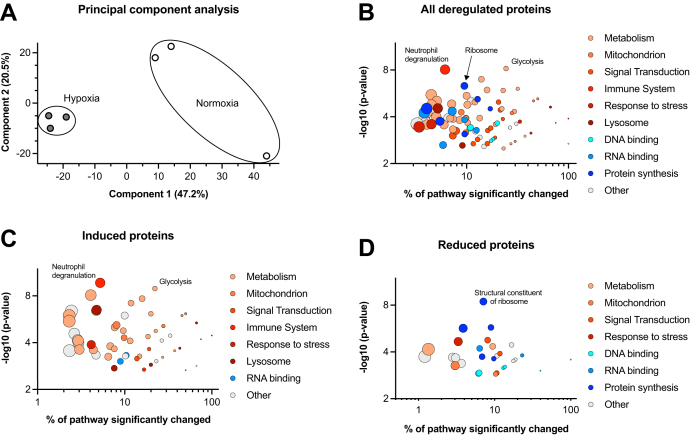
**Proteomic profiling reveals multiple changes associated with hypoxia.***A*, principal component analysis of the proteomic data showed a clear separation between normoxic and hypoxic fetal kidneys. *B*–*D*, functional annotation clustering of all significantly deregulated proteins (*B*), induced proteins (*C*), and reduced proteins (*D*) The significantly changed fraction of a pathway (x-axis) is plotted against its significance of enrichment (y-axis). The size of each point encodes the total number of members in that pathway. Induced proteins showed an enrichment of metabolic processes (glycolysis), mitochondrial or lysosomal proteins, and proteins involved in immune system response (neutrophil degranulation), whereas reduced proteins are enriched for DNA- and RNA-binding pathways (structural constituent of ribosome).

To elucidate the molecular pathways that underlie hypoxia-driven IUGR, freshly isolated kidneys from hypoxic or normoxic E18.5 fetuses were subjected to bottom-up proteome profiling using a nano-LC system (Dionex UltiMate 3000 RSLC) coupled to a high-resolution orbitrap mass spectrometer (Thermo QExactive). Principal component analysis showed striking differences between the hypoxic and normoxic kidneys (Fig. 2*A*). In total, 6307 proteins were identified (FDR < 0.01, supplemental Table S2) of which 436 were significantly deregulated (FDR < 0.05); 284 with increased abundance and 152 with decreased abundance. Functional annotation clustering using gene ontology (18, 19, 20) revealed an enrichment of specific mitochondrial, lysosomal, RNA-, and DNA-binding proteins, as well as proteins involved in specific metabolic processes or innate immune responses (Fig. 2, *B*–*D*). We categorized these proteins with regard to (1) nephron formation, (2) metabolic adaptation, and (3) accelerated aging, as presented and discussed in the following paragraphs.

### Repressed DNA Replication and Protein Synthesis Contribute to the Constrained Formation of New Nephrons in Chronic Hypoxia

**Fig. 3:**
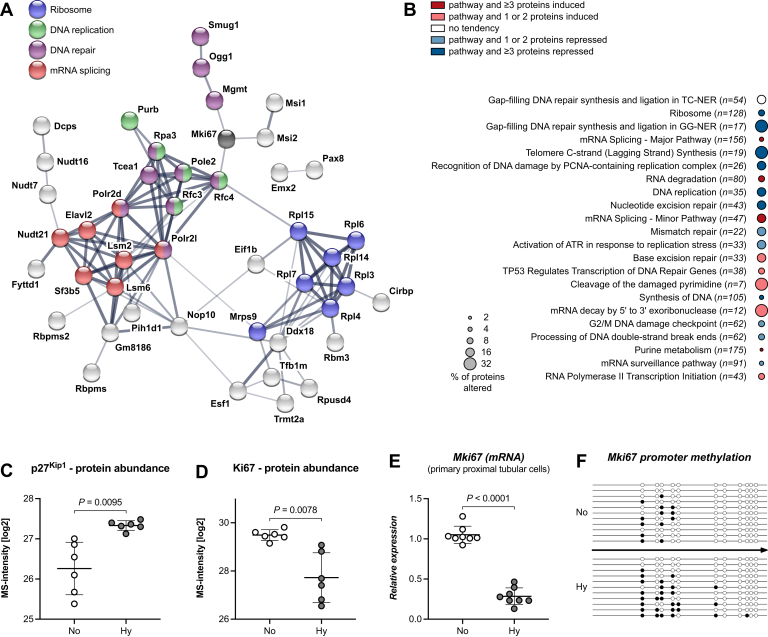
**The formation of new nephrons in hypoxia is associated with repressed DNA replication and protein synthesis.***A*, protein interaction network of significantly changed DNA- and RNA-binding proteins derived from the STRING database shows several protein clusters: ribosomal proteins (*blue*), proteins involved in DNA replication (*green*), DNA repair (*purple*), and mRNA splicing (*red*). The proliferation marker Ki67 (Mki67) is marked in *black*, highlighting its close relationship to DNA repair and DNA replication. *B*, a selection of pathways of significantly changed DNA- and RNA-binding proteins from KEGG and Reactome databases that exhibited the most prominent alterations in hypoxic kidneys, depicted in decreasing order of significance. RNA splicing and RNA degradation processes were enriched (*red*), while RNA translation, DNA replication, and repair pathways were repressed (*blue*). Only processes with an FDR <0.05 are shown. *C* and *D*, the cell cycle inhibitor p27^Kip1^ was enhanced (unpaired two-tailed *t* test, Welch’s correction, *p* = 0.0095), whereas the expression of the proliferation marker Ki67 was reduced (unpaired two-tailed *t* test, Welch’s correction, *p* = 0.0078) in hypoxic fetal kidneys. *E*, hypoxia reduced the mRNA expression level of *Mki67* in mouse primary proximal tubular cells (unpaired two-tailed *t* test, *p* < 0.0001). *F*, this reduction was mediated by hypermethylation of the *Mki67* promoter in hypoxic fetal kidneys. *Open circle* unmethylated, *black circle* methylated (Fisher's exact test, *p* = 0.0016; Mann–Whitney U-test, *p* = 0.0431).

Adverse events during development such as chronic fetal hypoxia have frequently been shown associated with reduced nephron numbers (27, 29, 30, 31, 32). Yet, an underlying mechanism convincingly explaining this finding could rarely be demonstrated. Clustering all 64 deregulated proteins belonging to the GO-terms “DNA-binding” and “RNA-binding” (a heatmap is shown in supplemental Fig. S2) using the STRING database (22) revealed multiple subnetworks including DNA repair, DNA replication, mRNA splicing, and ribosomal proteins (Fig. 3*A*). A more comprehensive list of enriched processes or pathways is shown in Figure 3*B* (FDR < 0.05). Of these “Ribosome” and “DNA replication” were among the top terms of repressed proteins, whereas mRNA splicing and “RNA degradation” were among the top terms of induced proteins. DNA repair processes showed a bipartite expression pattern. Here, proteins involved in nucleotide excision or mismatch repair were repressed, but those mediating “Base excision repair” were induced. Furthermore, the abundance level of the cell cycle inhibitor p27^Kip1^ was twofold increased (Fig. 3*C*) and that of the proliferation marker Ki67 was 3.4-fold reduced (Fig. 3*D*), which together indicate a slowdown of the cell division process. In particular, the mRNA expression level of *Mki67*, the gene encoding for Ki67, was significantly repressed in mouse primary proximal tubular cells cultured under hypoxic conditions (Fig. 3*E*). This repression was mediated through hypermethylation of the *Mki67* promoter region in hypoxic fetal kidneys (Fig. 3*F*). Thus, in fetal hypoxic kidneys, DNA synthesis, mRNA translation, and most DNA repair processes seem to be turned down, reducing the ability of the cells to grow and proliferate. In aggregate here, for the first time, proteome profiling data provide molecular evidence potentially explaining the diminished formation of nephrons.

### The Innate Immune System and Oxidative Stress Are Activated in Chronic Hypoxic Conditions

**Fig. 4:**
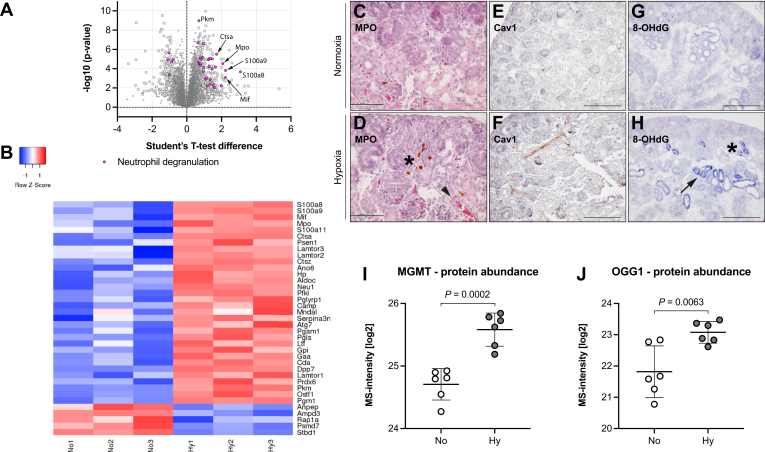
**Proteins involved in inflammatory response and oxidative stress are enriched in newly forming nephrons under hypoxia.***A*, depiction of all 34 significantly changed proteins for the annotation termed neutrophil degranulation (*pink circles*) among all significant (*larger gray circles*) and not significant (*smaller gray circles*) proteins of our data set. 29 had a higher abundance level, whereas five were reduced. *B*, a heatmap showing all *pink* proteins in (*A*) and their induction or repression in hypoxia, depicted in decreasing order of protein abundance. *C*–*H*, representative immunohistochemical images of E18.5 normoxic or hypoxic kidneys showing the infiltration of neutrophils and oxidative stress. *C* and *D*, immunohistochemistry for the neutrophil marker myeloperoxidase (MPO) revealed a clustering of these cells in the proximity of newly forming nephrons (*asterisk*) and adjacent to renal blood vessels (*arrowhead*) of hypoxic fetal kidneys (*D*). In normoxic controls (*C*), MPO-positive cells were rarely present. (Scale bars 100 μm). *E* and *F*, immunohistochemistry depicting enhanced expression of caveolin-1 (CAV1) in renal blood vessels of hypoxic fetal kidneys (*F*) compared with controls (*E*) (Scale bars 200 μm). *G* and *H*, 8-hydroxy-2′-deoxyguanosine (8-OHdG), a marker for oxidative DNA damage, was prominently enhanced in newly forming nephrons (*asterisk*) in the renal cortex and in cells of the proximal tubules (*arrow*) of hypoxic kidneys (*H*), whereas only few cells were stained in normoxic tissue (*G*) (Scale bars 200 μm). *I* and *J*, this increase in DNA damage occurred despite a simultaneous rise in O-6-methylguanine-DNA methyltransferase (MGMT; unpaired two-tailed *t* test, *p* = 0.0002) (*I*) and 8-oxoguanine glycosylase (OGG1; unpaired two-tailed *t* test, *p* = 0.0063) (*J*), two enzymes involved in the repair of oxidized DNA.

One of the most enriched terms appearing in the functional annotation analysis of all 436 proteins and the topmost for induced proteins was “Neutrophil degranulation” (Fig. 2, *B* and *C*), indicating an ongoing activation of the innate immune system in hypoxic fetal kidneys. Of the 34 associated proteins, 29 were induced and five repressed (pink in Fig. 4*A*). Among the induced proteins were many glycolytic and lysosome-related enzymes, several S100A protein family members, the inflammatory cytokine macrophage migration inhibitory factor (MIF), as well as the primary and secondary granule proteins neutrophilic granule protein (NGP), myeloperoxidase (MPO), Cathelicidin (CAMP), peptidoglycan recognition protein 1 (PGLYRP1), and lactoferrin (LTF). To verify the anticipated invasion of neutrophils in hypoxic fetal kidneys and to uncover their location within the tissue, renal sections were stained for MPO. MPO positive cells formed clusters in the nephrogenic zone of the renal cortex, in the proximity of blood vessels, adjacent to proximal tubuli, and occasionally could be found in medullary regions of hypoxic samples (Fig. 4*D* and supplemental Fig. S3, *A*–*C*). In contrast, normoxic fetal kidneys did not show infiltration or accumulation of neutrophils (Fig. 4*C*). Caveolin-1 (CAV1) was shown to enhance the transcellular migration of immune cells (33). Accordingly CAV1 was induced in hypoxic fetal kidneys (supplemental Fig. S3*D*), showing enhanced staining of renal blood vessels including those traversing the cortical region of the fetal kidney (Fig. 4, *E* and *F*). Degranulation of neutrophils produces a local burst of reactive oxygen species leading to increased oxidative stress and tissue damage including DNA oxidation. An important marker of DNA oxidation is 8-hydroxy-2′-deoxyguanosine (8-OHdG), which was enhanced in proximal tubules and in the nephrogenic zone of hypoxic fetal kidneys (Fig. 4, *G* and *H*), in the vicinity of neutrophil clustering. This rise in 8-OHdG damaged DNA occurred despite a simultaneous induction of MGMT and OGG1 (Fig. 4, I and *J*), two enzymes responsible for the removal of 8-OHdG. These results demonstrate an ongoing activation of the innate immune system in the developing kidney, which causes even more tissue damage in addition to the hypoxic stress. Along with the ineffective repair of these tissue lesions, a vicious circle might be initiated that further impairs nephrogenesis.

### Metabolic Adaptations to Hypoxia Result in Pronounced Glycolysis in the Fetal Kidney

**Fig. 5:**
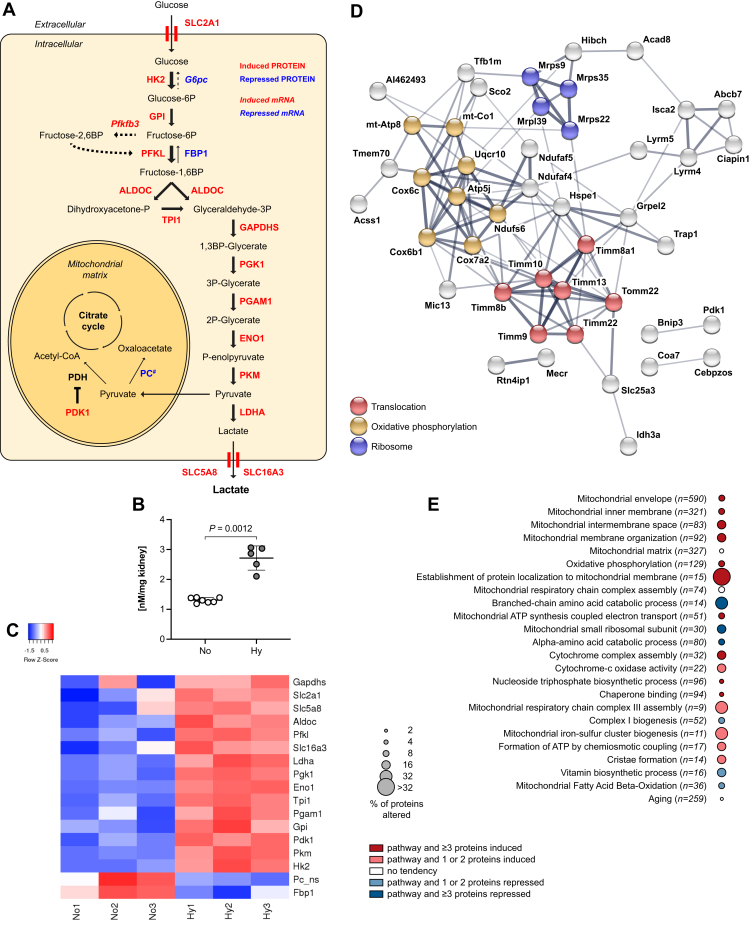
**Metabolic adaptations to hypoxia include enhanced glycolysis and compensatory mitochondrial protein import and respiratory chain assembly.***A*, a schematic depicting the significantly changed proteins that enhanced glycolysis in fetal hypoxic kidneys. Induced proteins are depicted in *red colors*, repressed proteins in *blue*, and not significantly changed proteins in *black* (*bold*). Enzyme substrates are shown in *black*. All enzymes involved in the conversion of glucose to pyruvate (glycolysis, numbered proteins ①–⑩ in *red*) and lactate dehydrogenase A (LDHA, *dark red*) were significantly enriched, whereas fructose-1,6-bisphosphatase 1 (FBP1), the antagonistic enzyme of 6-phosphofructokinase (③ – PFKL), was repressed. These changes mediate an enhanced flux of glucose to lactate. Furthermore, the glucose transporter 1 (SLC2A1, *orange*) and the monocarboxylate transporters SLC5A8 and SLC16A3 (both *orange*) were also significantly induced, promoting an increased transport of glucose into and lactate out of the cell, respectively. The utilization of pyruvate in the citrate cycle was repressed by either enhanced expression of pyruvate dehydrogenase kinase 1 (PDK1, *dark red*) that inhibits the PDH complex, or reduced expression of pyruvate carboxylase (PC, 1.64-fold decrease; ns, not significant). *B*, consequently, the concentration of lactate in the fetal renal tissue was increased in hypoxia (unpaired two-tailed *t* test, Welch’s correction, *p* = 0.0012). *C*, a heatmap showing all proteins in (*A*) and their induction or repression in hypoxia, depicted in decreasing order of protein abundance. *D*, protein interaction network of the significantly changed mitochondrial proteins derived from the STRING database shows several proteins clusters: mitochondrial ribosomal proteins (*blue*), proteins involved in oxidative phosphorylation (*orange*), or translocation (*red*). *E*, a selection of pathways of significantly changed mitochondrial proteins derived from GO, KEGG, and Reactome databases that exhibited the most prominent alterations in hypoxic kidneys, depicted in decreasing order of significance. Only processes with an FDR <0.05 are shown.

Chronic hypoxia is a severe condition to which cells have to adapt *via* metabolic changes in order to survive. Foremost to this is the maintenance of cellular ATP production that in the absence of sufficient oxygenation requires a shift from oxidative phosphorylation to glycolysis. Of all terms resulting from the functional annotation, “glycolytic process” was the most significant (Fig. 2*B*). All ten enzymes (red ①–⑩ in Fig. 5*A*) required for the conversion of glucose to pyruvate were induced in fetal hypoxic kidneys compared with normoxic controls (a heatmap is shown in Fig. 5*C*). Furthermore, the expressions of glucose transporter 1 (SLC2A1, orange in Fig. 4*A*) and lactate dehydrogenase A (LDHA, dark red in Fig. 5*A*) were also enhanced, which should facilitate an increased uptake of glucose into the cell and the augmented reduction of pyruvate to lactate, respectively. The alternative utilization of pyruvate in the citric acid cycle seemed to be impeded (1) by enhanced expression of pyruvate dehydrogenase kinase 1 (PDK1, dark red in Fig. 5*A*), which inactivates the pyruvate dehydrogenase complex in mitochondria and thus the oxidation of pyruvate to acetyl-CoA; and (2) by reduced levels of pyruvate carboxylase (PC), which catalyzes the conversion of pyruvate to oxaloacetate. On the other hand, fructose-1,6-bisphosphatase 1 (FBP1, blue in Fig. 5*A*) was reduced, further augmenting the potential flux of glucose toward pyruvate. All these enzymatic changes favor the production of lactate, and indeed, lactate concentration was increased in hypoxic fetal kidneys (Fig. 5*B*). Enhanced lactate production may lead to an unfavorable acidification. Yet, we found among the enriched proteins the monocarboxylate transporters SLC16A3 and SLC5A8 (orange in Fig. 5*A*), which are known to excrete lactate into the extracellular space to avoid toxic effects of cytoplasmic acidification. Thus, our model demonstrates the remarkable capability of fetal kidneys to adapt to chronic hypoxia by increasing glycolytic activity, which ensures sufficient ATP production and survival under this adverse condition.

### Fetal Hypoxia Promotes Compensatory Mitochondrial Protein Import and Respiratory Chain Assembly

Besides enhanced glycolysis, we found multiple alterations in mitochondrial protein abundance levels (a heatmap is shown in supplemental Fig. S4). Generation of protein networks using STRING revealed several clusters comprising proteins involved in translocation of proteins into mitochondria, oxidative phosphorylation, and mitochondrial ribosomal proteins (Fig. 5, *D* and *E*). Of note, multiple proteins linked to the inner mitochondrial membrane were induced, the site where oxidative phosphorylation (OXPHOS) takes place. OXPHOS encompasses five multiprotein complexes arranged along the inner mitochondrial membrane. Among the induced proteins were components of OXPHOS complexes I (NDUSF6), III (UQCR10), IV (COX6B1, COX6C, and COX7A2), and V (ATP5J, MTATP8), but also UQCC3 and SCO2, which are required for the correct assembly and function of complex III and IV, respectively (Fig. 5*D* and supplemental Fig. S4). SDHC and SDHD, subunits of complex II, were also upregulated (1.6- and 2.5-fold, respectively), but did not reach statistical significance. Moreover, not only components of the respiratory chain were enriched in fetal hypoxic kidneys, but also a multitude of proteins mediating their import into mitochondria. This included members of the outer membrane translocase (TOM – TOMM22), and the inner membrane translocase complex TIM22 (TIMM22, TIMM9, and TIMM10) and the associated TIMM8-TIMM13-complex (Fig. 5*D* and supplemental Fig. S4). Among the 21 mitochondrial proteins with reduced abundance were four mitochondrial ribosomal proteins, as well as proteins involved in amino acid catabolism, vitamin biosynthesis, or fatty acid beta-oxidation (Fig. 5, *D* and *E*, and supplemental Fig. S4). These findings point toward potential mitochondrial dysfunction and apparent efforts to regenerate damaged proteins (34), despite enhanced glycolysis and overall reduced protein synthesis.

### Lysosomal Biogenesis and Autophagy Are Enhanced in Hypoxic Fetal Kidneys

**Fig. 6:**
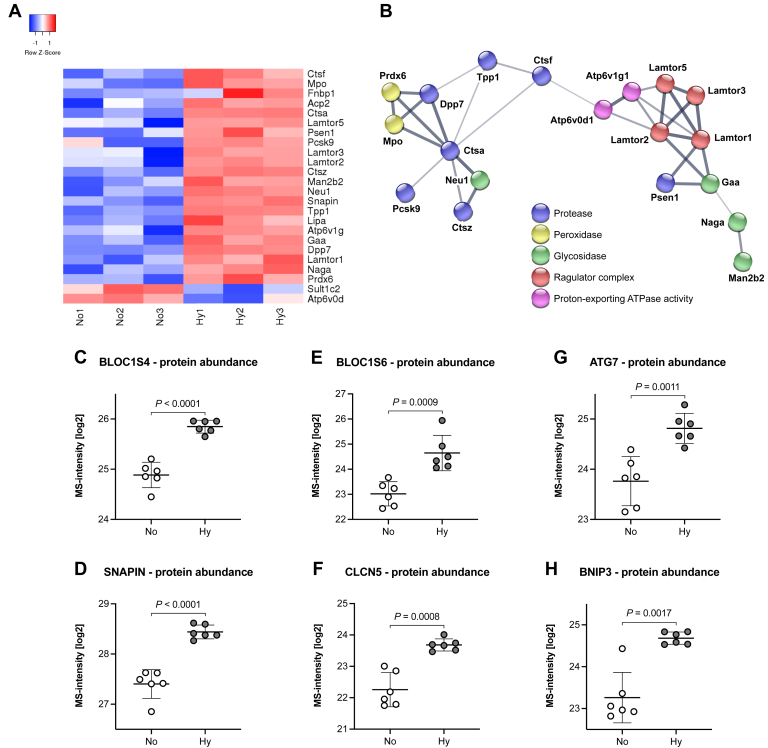
**Enhanced lysosomal biogenesis and autophagy in hypoxic fetal kidneys.***A*, a heatmap showing all significantly changed proteins for the GO term lysosome and their induction or repression in hypoxia, depicted in decreasing order of protein abundance. *B*, protein interaction network of the proteins in (*A*) derived from the STRING database, highlighting their functional relevance: proteases (*blue*), peroxidases (*yellow*), glycosidases (*green*), members of the regulator complex (*red*), and two members of proton pumps (*pink*). *C*–*E*, three of the eight structural components of the biogenesis of lysosome-related organelles complex 1 (BLOC1) were significantly induced in hypoxic kidneys. These were subunit 4 (BLOC1S4, unpaired two-tailed *t* test, *p* < 0.0001) (*C*), subunit 6 (BLOC1S6, unpaired two-tailed *t* test, *p* = 0.0009) (*D*), and subunit 7 (SNAPIN aka BLOC1S7; unpaired two-tailed *t* test, *p* < 0.0001) (*E*), suggesting an enhanced generation of lysosomes. *F*, another requirement in the ripening process of endosomes to lysosomes is their acidification. The proton chloride exchange transporter 5 (CLCN5), which is crucial for this step, was also upregulated in hypoxic kidneys (unpaired two-tailed *t* test, Welch’s correction, *p* = 0.0008). *C*, *G*, and *H*, lastly, several proteins involved in autophagy were induced under hypoxia. These included SNAPIN (*C*), autophagy related 7 (ATG7; unpaired two-tailed *t* test, *p* = 0.0011) (*G*), and BCL2/adenovirus E1B 19 kDa protein-interacting protein 3 (BNIP3; unpaired two-tailed *t* test, Welch’s correction, *p* = 0.0017) (*H*).

The lysosome was the second organelle that seems to be enriched under hypoxic conditions. However, in comparison to mitochondria, where 36% of the proteins were reduced, almost all lysosome-related proteins were upregulated (Fig. 6, *A* and *B*). Among them were nine lysosomal acid hydrolases, representing nearly 20% of the lysosomal acid hydrolases annotated in the KEGG pathway for lysosomes: four proteases (CTSA, CTSF, CTSZ, TPP1), three glycosidases (GAA, NAGA, NEU1), the lysosomal acid phosphatase 2 (ACP2), and the lysosomal acid lipase A (LIPA). Furthermore, we found evidence for increased biogenesis of lysosomes and related organelles. Three of the eight BLOC1 (biogenesis of lysosome-related organelles complex 1) components were significantly induced (Fig. 6, *C*–*E*), as well as the H/Cl exchange transporter 5 (CLCN5), which is a crucial player in the acidification of endosomes (Fig. 6*F*). Of note, Snapin (BLOC1 subunit 7) also plays a role in lysosomal acidification and in autophagosome maturation and function. Other induced proteins known to play a role in autophagy were Atg7 and Bnip3 (Fig. 6, *G* and *H*). Another requirement for autophagic flux is perinuclear clustering of lysosomes, mediated by the lysosomal Ragulator complex (35, 36) and two opposing motor protein families. Strikingly, four of the five members of the Ragulator scaffolding subunits (LAMTOR1, 2, 3, and 5) were significantly induced (Fig. 6, *A* and *B*); LAMTOR4 was also 1.81-fold induced, but did not reach statistical significance. Furthermore, kinesins, which mediate the outward movement of organelles, showed a tendency to be repressed, whereas dynein family members that facilitate the inward movement were induced, although not statistically significant (supplemental Fig. S5). Collectively these findings provide strong evidence that the housekeeping function of lysosomes is enhanced in fetal hypoxic kidneys, fully compatible with the anticipated need to renew damaged mitochondria.

### Premature Aging Exemplifies the Janus-Faced Aspects of Hypoxic Adaptation

**Fig. 7:**
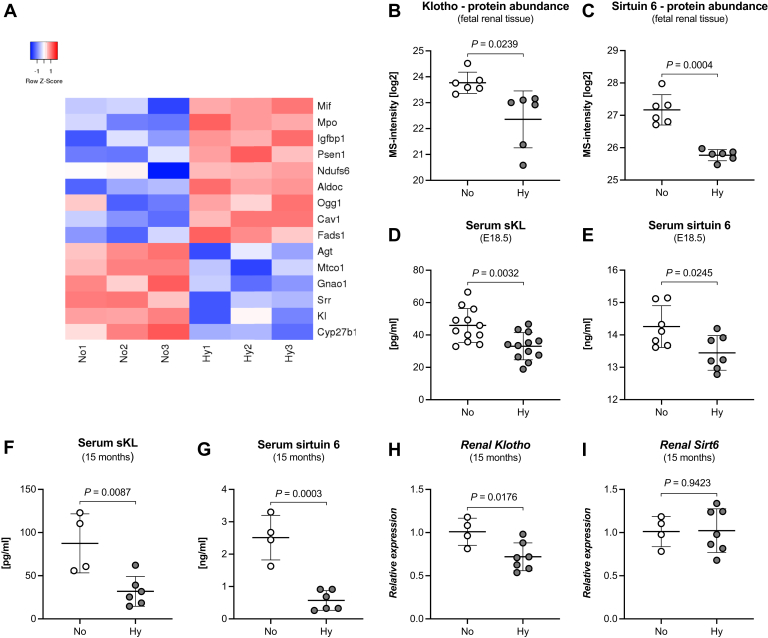
**Chronic hypoxia induces premature aging in fetal kidneys.***A*, a heatmap showing all significantly changed proteins for the GO term “Aging” and their induction or repression in hypoxia, depicted in decreasing order of protein abundance. *B* and *C*, the Abundance of the antiaging proteins klotho (*B*, unpaired two-tailed *t* test with Welch’s correction, *p* = 0.0239) and sirtuin 6 (*C*, unpaired two-tailed *t* test with Welch’s correction, *p* = 0.0004) was reduced in renal tissue from hypoxic fetuses. *D* and *E*, accordingly, serum levels of soluble Klotho (sKL) (*D*, unpaired two-tailed *t* test, *p* = 0.0032) and sirtuin 6 (*E*, unpaired two-tailed *t* test, *p* = 0.0245) were also reduced in hypoxic fetuses. *F* and *G*, these reductions of sKL (*F*, unpaired two-tailed *t* test, *p* = 0.0087) and sirtuin 6 (*G*, unpaired two-tailed *t* test, *p* = 0.0003) were still present in the serum of hypoxic offspring at 15 months. *H*, for klotho this seems to be due to reduced mRNA expression in the kidney (*H*, unpaired two-tailed *t* test, *p* = 0.0176). *I*, whereas renal Sirt6 mRNA expression was not changed between normoxic and hypoxic offspring at 15 months (unpaired two-tailed *t* test, *p* = 0.9423).

Opposed to the compensatory repair and rejuvenation mechanisms, we found 15 of the deregulated proteins belonging to the GO term “Aging” (Fig. 7*A*), representing the third category of hypoxic adaptations: accelerated aging. While most of these proteins play a role in one of the processes described above: neutrophil degranulation and lysosomes (MIF, MPO, PSEN1), mitochondria (NDUFS6, FADS1, MTCO1, CYP27B1), glycolysis (ALDOC), and DNA repair (OGG1); some have been described to directly effect on the life span of mice. In particular, the protein abundance of both klotho and sirtuin 6 was decreased in E18.5 hypoxic fetal kidneys (Fig. 7, *B* and *C*). Sirtuin 6 is a ubiquitously expressed enzyme with protein deacetylase and mono-ADP ribosyltransferase activity involved in the regulation of several cellular functions, including inflammation, glycolysis, and DNA repair. Its knockout leads to severe progeria in mice with a reduced life span of 1 to 3 months (37, 38). The kidney is the major site of klotho synthesis (*i.e.*, distal convoluted tubule—DCT contributes the majority of the protein with additional synthesis in the proximal convoluted tubules), where it acts locally as a membrane-bound beta-glucuronidase. The reduced abundance of klotho seems to be a specific process, since other proteins of the DCT including CALB1 were not altered. Klotho is also secreted into the circulation either by cleavage of the extracellular part of the membrane-bound form or by translation of an alternative splice variant. The level of circulating klotho (sKL) decreases with age (39, 40), which prompted us to assess its concentration and that of sirtuin 6 in trunk blood of hypoxic or normoxic E18.5 fetuses. Indeed, the concentrations of sKL and sirtuin 6 were significantly reduced in hypoxic E18.5 fetuses (Fig. 7, *D* and *E*). Furthermore and importantly, serum levels of klotho and sirtuin 6 were also still significantly reduced in aged mice (Fig. 7, *F* and *G*), indicating a permanent reduction of these two proteins throughout life. For klotho this seems to be due to significantly reduced mRNA expression levels in the kidneys of 15-month-old hypoxic offspring (Fig. 7*H*). However, renal mRNA expression levels of sirtuin 6 were unchanged between aged normoxic and hypoxic mice (Fig. 7*I*). Furthermore, changes in DNA methylation have been shown to be one of the most important mechanisms not only for nephron progenitor cell renewal and differentiation (41), but also for klotho expression (42, 43). However, in contrast to the hypermethylation of *Mki67* (Fig. 3*F*), the klotho promoter was hypomethylated during chronic hypoxia (supplemental Fig. S6). The methylation pattern of *Sirt6* could not be determined. To the best of our knowledge, our IUGR model is the first to delineate a mechanism leading to a premature aging phenotype, through synergy of inflammatory damage, ineffective repair, altered metabolism, and reduced abundance of antiaging proteins taking place at birth already.

### The Reduction of Antiaging Proteins in Response to Chronic Hypoxia Is Conserved Between Mice and Men

**Fig. 8:**
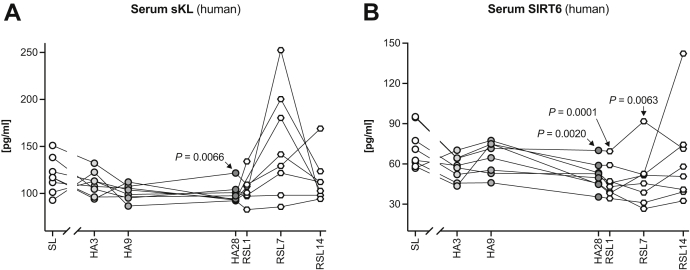
**The effect of chronic hypoxia on serum levels of klotho and sirtuin 6 is evolutionary conserved.** Human serum samples from a controlled study of nine healthy volunteers (eight males, one female) were collected 2 weeks before (sea level, SL), at three time points while at 3454 m (HA3, HA9, HA28), and 1, 7 and 14 days upon returning to SL (RSL1, RSL7, RSL14). *A*, human sKL concentrations quickly declined at HA and reached statistical significance at HA28 (RM one-way ANOVA with the Geisser–Greenhouse correction, Dunnett’s multiple comparisons test; all comparisons *versus* SL control group; *p* = 0.0066). Upon descent, sKL levels rapidly returned to SL levels or higher with a temporary peak around RSL7 (*p* = 0.2986). *B*, on the other hand, the serum concentrations of human SIRT6 were significantly repressed from H28 to RSL7 (RM one-way ANOVA with the Geisser–Greenhouse correction, Dunnett’s multiple comparisons test; all comparisons *versus* SL control group; for HA28: *p* = 0.002, for RSL1: *p* = 0.0001, for RSL7: *p* = 0.0063) and only started to rise again slowly at RSL14 (*p* = 0.7107).

In a last set of experiments, we asked whether the interplay between chronic hypoxia and reduced serum levels of antiaging proteins is an evolutionary conserved phenomenon. To this end, serum samples from a controlled study (10) of nine healthy volunteers (eight males, one female) collected 2 weeks before (sea level, SL), at three time points during an uninterrupted 28-day sojourn at 3454 m (high altitude, HA3, HA9, HA28), and 1, 7, and 14 days after their return to SL (RSL1, RSL7, RSL14) were analyzed for sKL and SIRT6 (Fig. 8). The samples obtained at SL served as controls for each participant. sKL and SIRT6 serum levels declined at high altitude and both reached statistical relevance at H28. Upon return to sea level, sKL increased to levels higher than before the stay at high altitude at RSL7 and returned to normal at RSL14 (Fig. 8*A*). On the other hand, SIRT6 continued to decline upon return to sea level and only started to incline again at RSL14 (Fig. 8*B*), suggesting differential regulation of these two antiaging proteins. In summary, these findings suggest that reduced serum klotho and sirtuin 6 levels may generally require exposure to chronic hypoxic conditions and may thus represent an evolutionary highly conserved process.

## Discussion

In the present study, we characterized multiple molecular changes that form a solid basis for Barker’s theory of fetal programming of adult diseases by combining a murine model of hypoxic IUGR with a bottom-up proteomic analysis, using the kidney as a readout. Major advantage of our analysis compared with other reports of hypoxia-induced IUGR mouse models (29, 30, 31, 32) is the presentation of a holistic dataset at the protein level of how chronic fetal hypoxia leads to reduced nephron formation, instead of focusing on a particular phenotype.

**Fig. 9:**
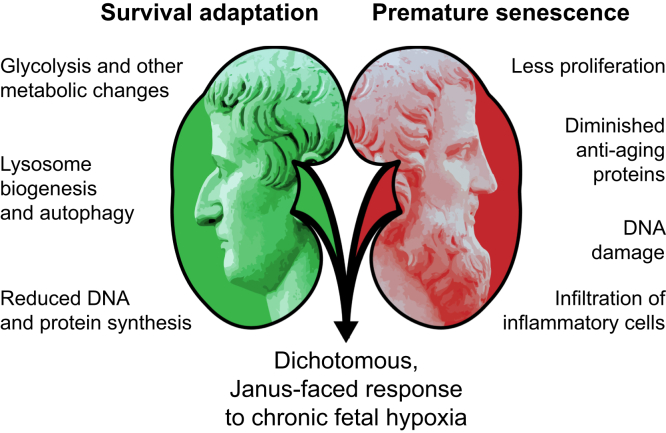
**Proposed model.** Chronic fetal hypoxia elicits a dichotomous, Janus-faced response in the developing kidney, comprising mechanisms that promote survival and others that induce accelerated aging.

A methodological limitation of the present approach is the use of total kidney lysates, precluding the allocation of the experimental observations to specific anatomic structures or cell types of the developing kidney. Other limitations include the general variability of outcomes among all published normobaric hypoxia mouse models with reported phenotypes ranging from altered Wnt signaling or aberrant expression of angiotensin II receptors to reduced GFR or proteinuria (27). This broad phenotype spectrum is most likely due to differences in the specific hypoxia protocols employed: particularly, variances in the severity of hypoxia and exposure time, the developmental stage at start of exposure or the requirement of a second hit to generate a phenotype, and the sex of the animals. Despite these differences, all studies, including ours, found the number of nephrons being reduced. Central theme of our findings is the dichotomous response to chronic fetal hypoxia categorized into rejuvenating and/or survival mechanisms and processes that promote premature aging (Fig. 9).

Chronic hypoxia induces enormous oxidative stress (44), while the concurrent lack of mitochondrial ATP formation impairs the regeneration of damaged proteins and organelles. This accumulation of damaged proteins (DAMPs) causes inflammasome activation, and subsequently, invading neutrophils are known to further damage functional epithelium. In the long-term, the proaging phenotype seems to prevail as chronic hypoxic kidneys exhibit multiple senescence-like hallmarks such as metabolic reprogramming, accumulation of mitochondrial and lysosomal mass, loss of proteostasis and mRNA processing, or persistent DNA damage (45). An increase in mitochondrial volume density with diminished respiratory capacity but enhanced efficiency of electron transport was also found in adult human skeletal muscle biopsies taken from participants of the high-altitude study (46, 47).

Furthermore, oxidative stress is also a main trigger of stress-induced premature senescence (SIPS) that occurs independently of telomere shortening, albeit sharing many downstream effectors of replicative senescence (48). Besides the p53-mediated activation of p21 and p16, increased levels of p27 also promote cell cycle arrest at G1/S due to the inhibition of CDK2-Cyclin E. G1-arrested cells exhibit heterochromatin changes, transcriptional alterations, and the senescence-associated secretory phenotype (SASP). SASP release is known to recruit leukocytes, promote an inflammatory response, but also to reduce cellular NAD levels that further restricts DNA repair processes and activity of sirtuins in neighboring, nonsenescent cells (49). Interestingly, the longevity gene *Sirt6*, responsible for more efficient DNA double-strand break repair in long-lived species (50), was shown to be reduced in senescent cells, preventing the proteasomal degradation of p27 (51). Furthermore, the most frequent oxidative DNA damage (8-OHdG) was also shown to accumulate with age (52, 53). Thus, our data point toward the establishment of a vicious circle downstream of hypoxia-driven persistent DNA damage that leads to the induction of SIPS, predetermining an aging phenotype in the renal tissue already during fetal development.

Importantly, this preprogrammed aging phenotype is not restricted to the kidney, but rather affects the entire organism, as reflected by reduced serum levels of klotho and sirtuin 6. In addition, the congruent results found in human serum samples upon long-term exposure to reduced oxygen levels strongly suggest that the downregulation of these antiaging protein in chronic hypoxia is evolutionary conserved between mouse and human. In this regard, three recent papers report reduced levels of klotho or SIRT6 in obstructive sleep apnea (54, 55) or in patients with interstitial lung abnormalities (56). Hence, insufficient tissue supply of oxygen at sea level leading to a hypoxic microenvironment may also promote a proaging phenotype despite normal overall oxygen availability. In a study published recently, we showed that global DNA hypomethylation represents a key regulatory event linking maternal nutrition during gestation and growth restriction in the kidney (41). Here, we specifically assessed the methylation patterns of murine *Mki67*, *Kl*, and *Sirt6*, and found differential, gene-specific methylation profiles despite less abundant levels of all three mammalian DNA methyltransferases (Dnmt1, Dnmt3a, and Dnmt3b) in our hypoxic proteomic dataset. The hypermethylation of *Mki67* illustrates a potential repressive mechanism that emphasizes the crucial connection between intrauterine hypoxia and a reduced nephron number at birth, implying that decreased proliferation is indeed a fundamental pillar underlying Barker’s and Brenner’s hypothesis. Moreover, it seems to be an actively controlled mechanism, since it takes place despite the overall reduction of Dnmt protein abundance. During fetal development, this process represents a survival advantage giving the fetus time to repair DNA damage. However this comes at a high cost—an increased risk for renal disease in adults.

In conclusion, based on a unique biomarker panel, we describe a solid foundation for Barker’s theory, delineating a dichotomous, Janus-faced response that comprises pathways that ensure survival, but also promote a senescence-like phenotype in response to chronic fetal hypoxia in the kidney. These distinct conditions apparently contribute to the restricted formation of nephrons at births, which forms the basis of accelerated renal function decline in adults, thus substantiating Brenner’s hypothesis. Furthermore, we partially corroborated our findings in chronic hypoxia exposure of adult humans, providing evidence that the repression of antiaging proteins is a highly conserved mechanism. By monitoring and targeting the processes that predetermine an aging phenotype in tissue or blood samples, novel interventions might be implemented to counteract chronic hypoxia-associated accelerated aging in human diseases.

## Data Availability

The mass spectrometry proteomics data have been deposited to the ProteomeXchange Consortium (http://proteomecentral.proteomexchange.org) *via* the PRIDE partner repository (15) with the dataset identifier PXD018999 and 10.6019/PXD018999. All other data supporting the findings of this study are available from the corresponding author on request.

## Supplemental data

This article contains supplemental data.

## Conflict of interest

The authors declare no competing interests.
