# Supplementary material for: Dichotomous Responses to Chronic Fetal Hypoxia Lead to a Predetermined Aging Phenotype
Source: Mol Cell Proteomics. 2021 Dec 24;21(2):100190. doi: 10.1016/j.mcpro.2021.100190 (PMC8808178; doi:10.1016/j.mcpro.2021.100190)
Supplement: Supplemental Figures S1–S6 and Table S1 [file mmc1.pdf]

Supplementary information for

Dichotomous responses to chronic fetal hypoxia lead to a predetermined aging phenotype

Authors and affiliations:

Stefan Rudloff[1,†], Andrea Bileck[2,†], Lukas Janker[2], Nicola Wanner[3], Nastassia Liaukouskaya[3], Carsten Lundby[4,5], Tobias B. Huber[3], Christopher Gerner[2,*] and Uyen Huynh-Do[1,*]

[1]Division of Nephrology and Hypertension, University of Bern and University Hospital Bern, Freiburgstrasse 15, CH-3010 Bern, Switzerland

[2]Department of Analytical Chemistry, Faculty of Chemistry, University of Vienna, Waehringer Strasse 38, A-1090 Vienna, Austria

[3]University Medical Center Hamburg-Eppendorf, III. Medizinische Klinik und Poliklinik, Martinistrasse 52, D-20246 Hamburg, Germany

[4]Centre for Physical Activity Research (CFAS), Rigshospitalet Section 7641, Ole Maaloesvej 24, DK-2100 Copenhagen, Denmark

[5]Faculty of Social and Health Sciences, Section for Health and Exercise Physiology, Inland Norway University of Applied Sciences, NO-2624 Lillehammer, Norway

†Authors contributed equally to this work

*corresponding authors:	Uyen Huynh-Do:	uyen.huynh-do@insel.ch
		+41 31 632 31 41
	Christopher Gerner:	christopher.gerner@univie.ac.at
		+43 1 4277 52302
		+43 1 4277 52302

Supplementary Materials

Supplementary Fig. S1.	Chronic fetal hypoxia causes intrauterine growth restriction.
Supplementary Fig. S2.	Deregulated DNA- and RNA-binding proteins.
Supplementary Fig. S3.	Neutrophils accumulate in hypoxic fetal kidneys.
Supplementary Fig. S4.	Deregulated proteins with mitochondrial function.
Supplementary Fig. S5.	Deregulated dynein and kinesin protein family members.
Supplementary Fig. S6.	Klotho promoter methylation.
Supplementary Table S1.	qPCR primers and probes.
Supplementary Table S2.	Protein identifications (separate file).
Supplementary Data Sets.	Source Date File S1 (separate file).

Supplementary Figures

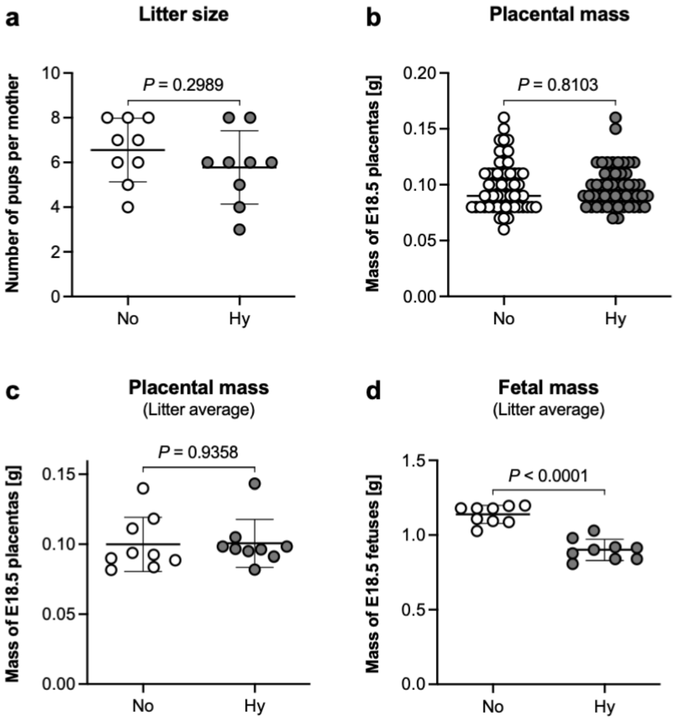

Supplementary Figure S1 Chronic fetal hypoxia causes intrauterine growth restriction. (**a**) Litter size (unpaired two-tailed t test, *P* = 0.2989) and (**b**) placental mass (unpaired two-tailed t test, *P* = 0.8103; N = 59 for normoxia and N = 52 for hypoxia) were comparable between normoxic (N = 9) and hypoxic gestations (N = 9). (**c**) Furthermore, also the litter average placental mass was unchanged (unpaired two-tailed t test, *P* = 0.9358). (**d**) On the contrary, the litter average of fetal was significantly reduced for hypoxic gestations (N = 9) compared to normoxic gestations (N = 9) (unpaired two-tailed t test, *P* < 0.0001).

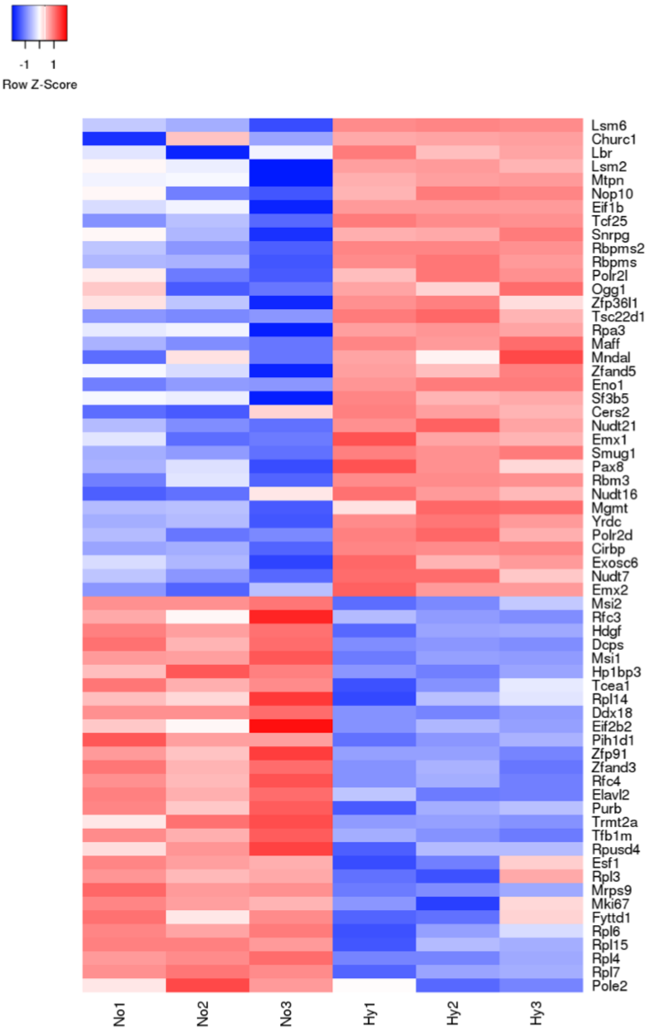

Supplementary Figure S2 Deregulated DNA- and RNA-binding proteins. A heatmap showing all significantly changed DNA- and RNA-binding proteins (n=64) and their induction or repression in hypoxia, depicted in decreasing order of protein abundance.

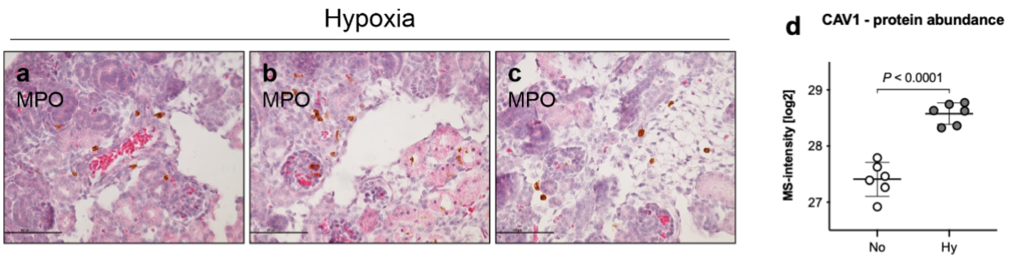

Supplementary Figure S3 Neutrophils accumulate in hypoxic fetal kidneys. (**A-C**) Immunohistochemistry for the neutrophil marker myeloperoxidase (MPO) revealed the clustering of these leukocytes in the vicinity of renal blood vessels (**A**), around newly forming nephrons and proximal tubules (**B**), but also in medullary regions of the hypoxic kidney (**C**). (**D**) The abundance of CAV1 was significantly enhanced in hypoxia (unpaired two-tailed t test, $P < 0.0001$).

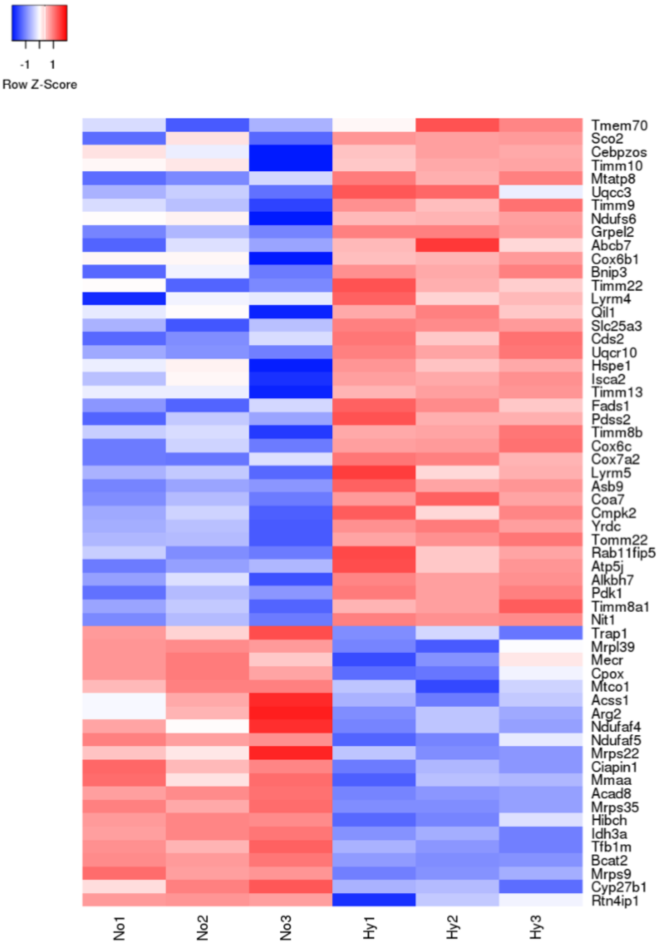

Supplementary Figure S4 Deregulated proteins with mitochondrial function. A heatmap showing all significantly changed mitochondrial proteins and their induction or repression in hypoxia, depicted in decreasing order of protein abundance.

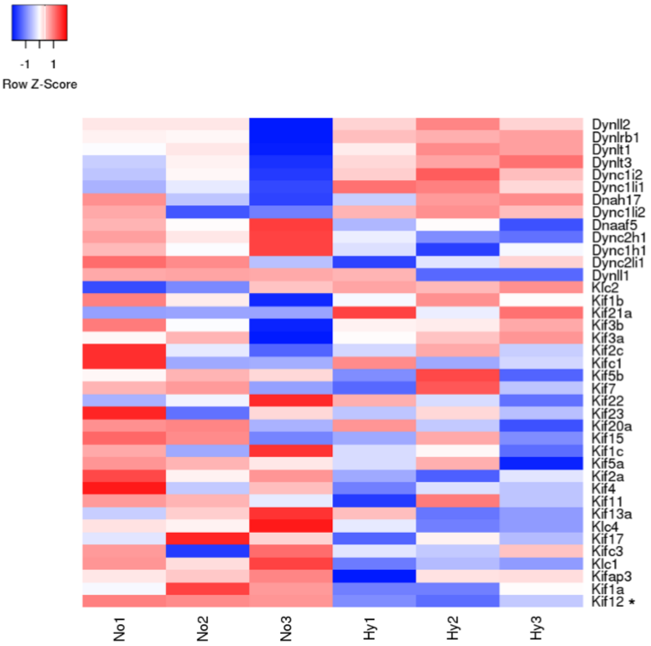

Supplementary Figure S5 Deregulated dynein and kinesin protein family members. Dyneins showed a tendency for increase abundance, whereas the majority of kinesins was repressed. Kif12 (asterisk) was the only protein to be significantly altered.

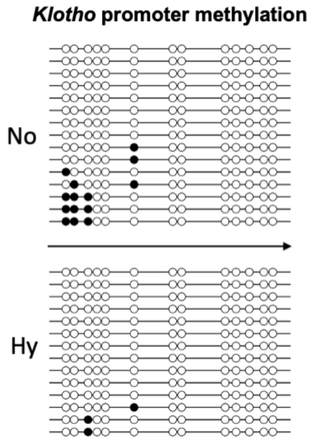

Supplementary Figure S6 *Klotho* promoter methylation. Under hypoxia, the *Klotho* promoter is hypomethylated. Open circle unmethylated, black circle methylated. (Fisher's exact test, P = 0.0117).

Supplementary Table

Table S1 qPCR primers and probes

Universal Probe Library (Roche)			
**Gene Symbol**	**Forward primer**	**Reverse Primer**	**Probe Number**
Col1a1	aggcaagcctggtgaaca	accagggaaacctctctcg	80
Col3a1	tggaccccaaggtcttcc	catctgatccagggtttcca	64
Mki67	gacaattgcaagtaactaattcagga	tccgagtactggatagcactttt	9
Ppia	acgccactgtcgcttttc	ctgcaaacagctcgaagga	46
Rplp0	actggtctaggacccgagaag	ctcccaccttgtctccagtc	9
Vim	gtaccggagacaggtgcagt	ttctcttccatctcacgcatc	1
